# Predictive model of bleeding following endoscopic sphincterotomy for the treatment of choledocholithiasis in hemodialysis patients: A retrospective multicenter study

**DOI:** 10.1002/jgh3.12363

**Published:** 2020-05-17

**Authors:** So Nakaji, Yoshihiro Okawa, Kenji Nakamura, Masahiro Itonaga, Masami Inase, Harutoshi Sugiyama, Rei Suzuki, Kenji Yamauchi, Hiroki Matsui, Nobuto Hirata, Junko Saito, Naoki Ishii, Toshio Tsuyuguchi, Hironari Kato, Masayuki Kitano, Naoya Kato, Hiromasa Ohira, Hiroyuki Okada, Takuji Torimura, Hiroyuki Maguchi

**Affiliations:** ^1^ Department of Gastroenterology Kameda Medical Center Chiba Japan; ^2^ Department of Gastroenterology Chikamori Hospital Kochi Japan; ^3^ Department of Gastroenterology St. Luke's International Hospital Tokyo Japan; ^4^ Second Department of Internal Medicine Wakayama Medical University Wakayama Japan; ^5^ Department of Gastroenterology Ebina General Hospital Ebina Japan; ^6^ Department of Gastroenterology, Graduate School of Medicine Chiba University Chiba Japan; ^7^ Department of Gastroenterology Fukushima Medical University Fukushima Japan; ^8^ Department of Gastroenterology and Hepatology Okayama University Graduate School of Medicine, Dentistry and Pharmaceutical Sciences Okayama Japan; ^9^ Clinical Research Support Division Kameda Institute for Health Science, Kameda College of Health Sciences Chiba Japan; ^10^ Division of Gastroenterology Tokyo Shinagawa Hospital Shinagawa City Japan; ^11^ Department of Medicine Kurume University School of Medicine Kurume Japan

**Keywords:** choledocholithiasis, hemodialysis, postendoscopic sphincterotomy bleeding

## Abstract

**Background and Aim:**

Although hemodialysis (HD) is a strong risk factor for postendoscopic sphincterotomy (ES) bleeding, additional risk factors in HD patients remain unclear. There is no model for predicting post‐ES bleeding risk in HD patients. Therefore, we conducted a retrospective multicenter study to reveal these risk factors and develop a predictive model of post‐ES bleeding in HD patients.

**Methods:**

We retrospectively reviewed the medical records of HD patients who underwent ES at eight hospitals between January 2006 and December 2016, with post‐ES bleeding as the main outcome measure. Univariate analyses were performed to extract possible risk factors for post‐ES bleeding. Factors that were clinically important and statistically significant in our univariate analyses were then included in our logistic regression analysis for the development of a multivariate predictive model of post‐ES bleeding. This predictive model was visualized using a predictive nomogram.

**Results:**

Post‐ES bleeding occurred in 20 (16.3%) of 123 HD patients. Based on clinically important factors and the results of our univariate analyses, platelet count, prothrombin time (international normalized ratio), and HD duration were included in our predictive model of post‐ES bleeding. Receiver operating characteristic analysis found that this model had an area under the curve of 0.715 (95% confidence interval, 0.609–0.822). We developed a predictive nomogram based on these results.

**Conclusions:**

We demonstrated that post‐ES bleeding is more common in HD patients than in the general population and succeeded in constructing a predictive model that can effectively identify HD patients at risk of post‐ES bleeding.

## Graphical abstract

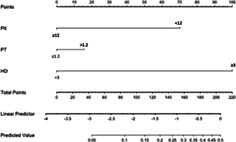

## Introduction

Because of the increasing number of patients undergoing hemodialysis (HD), the number of cases involving the treatment of choledocholithiasis in HD patients is also increasing. In many cases, endoscopic sphincterotomy (ES) at the papilla of Vater,^1^ which was developed by Kawai *et al*.^2^ and Classen *et al*.^3^ in the early 1970s, is chosen for treatment. Complications of this procedure include post‐ES bleeding in approximately 1–2% of patients, as well as post‐ES bleeding‐related death in 0.05% of cases.^4^, ^5^ In HD patients, the rate of post‐ES bleeding is greater than in the general population due to their increased bleeding tendency.^6^, ^7^, ^8^, ^9^, ^10^ This increased bleeding risk has been reported to be attributed to uremia‐induced platelet dysfunction and intermittent anticoagulant use during HD.^11^ However, post‐ES bleeding is caused not only by the direct influence of HD but also by other risk factors such as procedures associated with endoscopic retrograde cholangiopancreatography (ERCP) and other patient characteristics.

Despite the likely contribution of multiple risk factors, there is no model for predicting post‐ES bleeding risk in HD patients, with previous studies being too small to evaluate these factors. Thus, we conducted a retrospective multicenter study to identify additional risk factors and create a predictive model of post‐ES bleeding in patients with HD.

## Methods

### *Ethical statement*

This study received ethical approval from the research ethics committee of each hospital involved. All patients provided informed consent for their inclusion in this study.

### *Patient recruitment and data collection*

We retrospectively reviewed the medical records of patients who underwent ES at eight hospitals between January 2006 and December 2016. Patients were included if they were undergoing HD and had ES performed on naïve papillae and were excluded if they had a malignant biliary obstruction and/or did not undergo laboratory testing on the day of ES.

After enrollment in our study, the following data were collected from each patient: age; gender; platelet count and prothrombin time (international normalized ratio) (PT‐INR) on the day of ES; and the presence or absence of Child‐Pugh class C cirrhosis, a diverticulum, surgically altered upper gastrointestinal anatomy (excluding Billroth I anastomoses), and antithrombotic therapy (i.e. antiplatelet or anticoagulant therapy). Regarding antithrombotic therapy, patients were divided into two categories: low risk (i.e. no medication or adequate drug withdrawal) and high risk (i.e. inadequate drug withdrawal or heparinization). The required drug withdrawal periods were defined in accordance with Japanese guidelines.^12^ The following HD‐related data were also examined: the duration of HD and the antithrombotic drug administered during HD in the post‐ES period (i.e. unfractionated heparin [UFH], low‐molecular‐weight heparin [LMWH], or nafamostat mesilate [NM]). Concerning ERCP‐related procedures, the following data were collected: electrosurgical generator unit setting (i.e. automatically controlled blended current mode [i.e. ENDO CUT] or pure‐cut mode); whether endoscopic papillary large balloon dilation (EPLBD), precut sphincterotomy, and temporary biliary stenting were performed; and the presence or absence of bleeding during the procedure.

### *Hospital volume and the* *ES* *procedure*

Over 200 ERCP procedures were performed per year at each hospital. Furthermore, at two of the eight hospitals, over 500 ERCPs were performed per year. Regarding ES, over 50 procedures were performed per year at each hospital, with the number of procedures exceeding 200 at one hospital.

The ES procedure itself was carried out with a sphincterotome through a side‐viewing duodenoscope. For cases of surgically altered upper gastrointestinal anatomy (i.e. Billroth II or Roux‐en‐Y anastomoses), a forward‐viewing conventional endoscope, oblique‐viewing endoscope, or balloon endoscope was used. Incision length was based on stone size within the oral protrusion, and EPLBD was performed as necessary. For difficult cases with deep cannulation, precut sphincterotomy was carried out. For cases where stone removal in a single procedure was difficult, temporary biliary stenting was performed.

### *Definition of* *post‐ES* *bleeding*

Post‐ES bleeding was defined as clinically evident bleeding within 14 days, as set out in the consensus criteria proposed by Cotton *et al*.^1^ The severity of post‐ES bleeding was classified as follows: mild, where overt bleeding was combined with a reduction in the patient's hemoglobin level to <3 g/dL with no need for transfusion; moderate, where a blood transfusion of ≤4 units was needed in patients who did not require angiographic intervention or surgery; and severe, where a blood transfusion of ≥5 units, or angiographic or surgical intervention, was required.^1^

### *Statistical analysis and outcome measures*

All statistical analyses were performed using R 3.5.1, with the rms package used for cross‐validation.^13^ Continuous variables were compared between patients with and without post‐ES bleeding using the *t*‐test, whereas categorical variables were analyzed using the *χ* ^2^‐test or Fisher's exact test. *P*‐values of <0.05 were considered statistically significant.

#### *Calculation of required sample size*

We used post‐ES bleeding as the main outcome measure. The required sample size was calculated based on the hypothesis that post‐ES bleeding would have occurred in 25.5% of enrolled patients, which was determined from previous reports on post‐ES bleeding incidence.^6^, ^7^, ^8^, ^9^, ^10^ In addition, the error span and confidence level were set at 10% and 95%, respectively. Consequently, 73 HD patients were needed to perform our risk factor analyses.

### *Univariate analyses*

We performed univariate analyses with the following variables to extract possible risk factors for post‐ES bleeding in HD patients: age; gender; platelet count; PT‐INR; HD duration; antithrombotic agent administered during HD in the post‐ES period; electrosurgical generator unit setting; and the presence or absence of Child‐Pugh class C cirrhosis, a diverticulum, surgically altered upper gastrointestinal anatomy, antiplatelet therapy, anticoagulant therapy, EPLBD, precut sphincterotomy, bleeding during the procedure, and temporary biliary stenting. To account for the possibility of a nonlinear association between bleeding risk and continuous variables, we also compared the post‐ES bleeding rate between two groups defined by various cut‐off points for each continuous variable.

### *Multivariate analysis and predictive model development*

To develop a multivariate predictive model, we then performed logistic regression analysis with variables that were statistically significant in our univariate analyses or are important in clinical practice. We determined the cut‐off points for each of these continuous variables based on statistical significance in univariate analysis. The predicted probability of post‐ES bleeding was then estimated using our logistic regression model. The discriminative ability of the model was assessed by calculating the area under the curve in receiver operating characteristic analysis. Calibration of the model was assessed by plotting the actual probability of post‐ES bleeding against the model‐predicted probability. The predictive model was internally validated through cross‐validation with 1000 bootstrap samples to prevent overfitting and obtain a relatively unbiased estimate. Finally, to visualize the predictive model, we created a predictive nomogram.

### *Secondary outcome measures*

The following clinical parameters were described as secondary outcome measures: clinical outcome, post‐ES bleeding severity, the duration of hospitalization, the interval between ES and bleeding, the total number of hemostasis procedures, the initial hemostasis procedures, total blood transfusion requirement, and other ERCP‐related adverse events.

## Results

A total of 140 HD patients underwent ES for choledocholithiasis during the study period, 123 of whom met the inclusion criteria. Post‐ES bleeding occurred in 20 of these 123 patients (16.3%; 95% confidence interval [CI], 10.2–24.0%). Patient clinical characteristics are summarized in Table 1. The median age was 71 years (range: 47–101), platelet count was 153 000 (range: 23 000–503 000), PT‐INR was 1.08 (range: 0.83–1.68), and the duration of HD was 5 years (range: 1–24). Of patients, 26% were receiving antiplatelet therapy, and 13.8% were receiving anticoagulant therapy.

**Table 1:** Patient clinical characteristics

Factor	Group	*n*/median [range]	%
Total		123	
Age		71 [47–101]	
Gender	F	44	35.8
	M	79	64.2
Platelet count ×10 000		15.3 [2.3–50.3]	
PT‐INR		1.08 [0.83–1.68]	
Child‐Pugh class C cirrhosis	−	123	100
Diverticulum	−	79	64.2
	+	44	35.8
Surgically altered upper GI anatomy	−	120	97.6
	+	3	2.4
Antiplatelet drugs	No medication	91	74
	Adequate drug withdrawal	28	22.8
	Inadequate drug withdrawal	4	3.3
Anticoagulant drugs	No medication	106	86.2
Warfarin	Adequate drug withdrawal	4	3.3
	Heparinization	10	8.1
DOAC	Inadequate drug withdrawal	3	2.4
Cut mode	Endo cut	115	93.5
	Pure cut	8	6.5
Precut sphincterotomy	−	113	91.9
	+	10	8.1
EPLBD	−	106	86.2
	+	17	13.8
Temporary biliary stenting	−	97	78.9
	+	26	21.1
Anticoagulant drugs during HD	NM	79	64.2
	LMWH	14	11.4
	UFH	30	24.4
Duraion of HD (years)		5 [1–24]	
Bleeding during procedure	−	95	77.2
	+	28	22.8

DOAC, direct oral anticoagulants; EPLBD, endoscopic papillary large balloon dilation; GI, gastrointestinal; HD, hemodialysis; LMWH, low‐molecular‐weight heparin; NM, nafamostat mesilate; PT‐INR, international normalized ratio of prothrombin time; UFH, unfractionated heparin.

### *Univariate analyses*

The results of our univariate analyses are shown in Table 2. PT‐INR was significantly different between patients with and without post‐ES bleeding (*P* = 0.048), but other factors do not show significant differences. Table 3 shows the results of our comparison of post‐ES bleeding rates between two groups defined by various cut‐off points for each continuous variable. Platelet count of <120 000 and <140 000 and the duration of HD of <3 years have strong significance (*P* = 0.005, 0.046, and 0.025, respectively). Regarding PT‐INR, the bleeding rate among the groups showed a trend of increase correlating with the increase in PT‐INR (≤1.15, >1.15: 14.8% *vs* 20%, ≤1.2, >1.2: 14.7% *vs* 23.8%, ≤1.25, >1.25: 15.0% *vs* 30%, and ≤1.3, >1.3: 14.7% *vs* 42.9%, respectively) but did not show significance statistically. Based on factors that are important in clinical practice and were significant in our univariate analyses, we included platelet count, PT‐INR, and the duration of HD in our development of a predictive model of post‐ES bleeding. The cut‐off points for these variables were 120 000/mL and 1.2 and 3 years, respectively.

**Table 2:** Univariate analyses comparing clinical characteristics between patients with and without postendoscopic sphincterotomy bleeding

Factor	Group	Post‐ES bleeding		Univariate analysis
		−	+	*P*‐value
*n*		103	20	
Age, mean ± SD		72.50 ± 11.22	71.85 ± 9.54	0.807
Gender (%)	F	38 (86.4)	6 (13.6)	0.0619
	M	65 (82.3)	14 (17.7)	
Platelet count ×10 000, mean ± SD		17.71 ± 7.89	15.03 ± 7.40	0.162
PT‐INR, mean ± SD		1.09 ± 0.13	1.15 ± 0.15	0.048
Diverticulum (%)	−	68 (86.1)	11 (13.9)	0.445
	+	35 (79.5)	9 (20.5)	
Surgically altered upper GI anatomy (%)	−	100 (83.3)	20 (16.7)	1
	+	3 (100)	0 (0)	
Antiplatelet drugs (%)	Low risk	99 (83.2)	20 (16.8)	1
	High risk	4 (100)	0 (0)	
Anticoagulant drugs (%)	Low risk	95 (84.1)	18 (15.9)	0.665
	High risk	8 (80)	2 (20)	
Cut mode (%)	Endo cut	95 (82.6)	20 (17.4)	0.351
	Pure cut	8 (100)	0 (0)	
Precut sphincterotomy (%)	−	94 (76.4)	19 (13.6)	1
	+	9 (90)	1 (10)	
EPLBD (%)	−	88 (83.0)	18 (17.0)	0.737
	+	15 (88.2)	2 (11.8)	
Temporary biliary stenting (%)	−	80 (82.5)	17 (17.5)	0.563
	+	23 (88.5)	3 (11.5)	
Bleeding during procedure	−	80 (84.2)	15 (15.8)	0.776
	+	23 (82.1)	5 (17.9)	
Anticoagulant drugs during HD (%)	NM	69 (87.3)	10 (12.7)	0.143
	LMWH	9 (64.3)	5 (35.7)	
	UFH	25 (83.3)	5 (16.7)	
Duraion of HD, mean ± SD (years)		6.74 ± 5.48	7.60 ± 5.71	0.524

EPLBD, endoscopic papillary large balloon dilation; GI, gastrointestinal; HD, hemodialysis; LMWH, low‐molecular‐weight heparin; NM, nafamostat mesilate; PT‐INR, international normalized ratio of prothrombin time; UFH, unfractionated heparin.

**Table 3:** Comparison of postendoscopic sphincterotomy bleeding rate between two groups defined by various cut‐offs and multivariate analysis including platelet count (<12, ≥12), prothrombin time (international normalized ratio) (≤1.2, >1.2), and HD duration (<3, ≥3)

Factor	Group	*n* (%)		Univariate analysis	Multivariate analysis		
		Non‐bleeding	Bleeding	*P*‐value	OR	95% CI	*P*‐value
Age (years)	<65	24 (82.6)	5 (17.4)	1			
	65≤	79 (84.0)	15 (16.0)				
	<70	43 (82.7)	9 (17.3)	0.809			
	70≤	60 (84.5)	11 (15.6)				
	<75	62 (84.9)	11 (15.1)	0.804			
	75≤	41 (82)	9 (18)				
	<80	77 (84.6)	14 (15.4)	0.781			
	80≤	26 (81.3)	6 (18.7)				
Platelet count ×10 000	<10	16 (76.2)	5 (23.8)	0.333			
	10≤	87 (85.3)	15 (14.7)				
	<12	23 (67.6)	11 (32.4)	0.005	Reference		
	12≤	80 (89.9)	9 (10.1)		0.277	0.098–0.782	0.0154
	<14	36 (75)	12 (25)	0.046			
	14≤	67 (89.3)	8 (10.7)				
	<16	49 (79.0)	13 (20.2)	0.222			
	16≤	54 (88.5)	7 (11.5)				
PT‐INR	≤1.15	75 (85.2)	13 (14.8)	0.589			
	1.15<	28 (80)	7 (20)				
	≤1.2	87 (85.3)	15 (14.7)	0.333	Reference		
	1.2<	16 (76.2)	5 (23.8)		1.330	0.381–4.660	0.654
	≤1.25	96 (85.0)	17 (15.0)	0.207			
	1.25<	7 (70)	3 (30)				
	≤1.3	99 (85.3)	17 (14.7)	0.084			
	1.3<	4 (57.1)	3 (42.9)				
Duration of HD (years)	<2	17 (100)	0 (0)	0.072			
	2≤	86 (81.1)	20 (19.9)				
	<3	29 (96.7)	1 (4.3)	0.025	Reference		
	3≤	74 (80.0)	19 (20.0)		6.220	0.779–49.600	0.0847
	<4	38 (88.4)	5 (11.6)	0.443			
	4≤	65 (81.3)	15 (18.7)				
	<5	46 (86.8)	7 (13.2)	0.469			
	5≤	57 (81.4)	13 (18.6)				

CI, confidence interval; HD, hemodialysis; OR, odds ratio; PT‐INR, international normalized ratio of prothrombin time.

### *Multivariate analysis and the predictive model*

**Figure 1:**
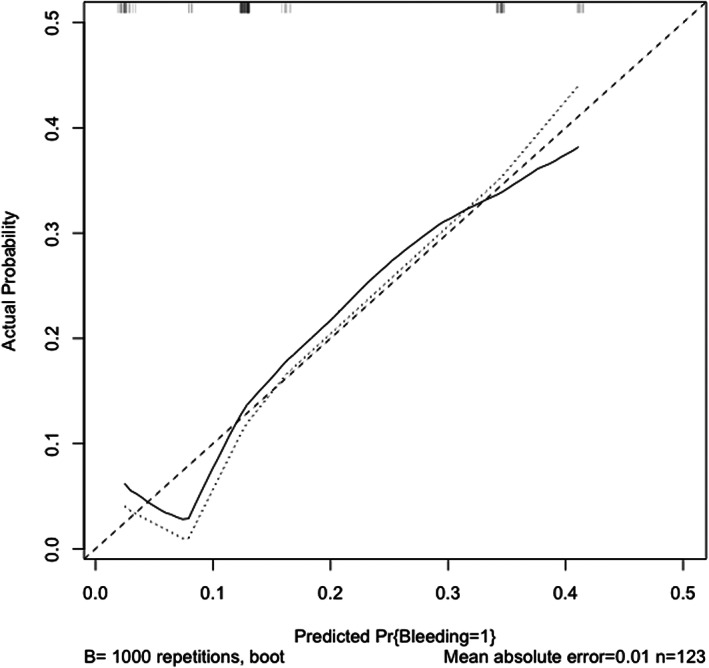
Calibration plots for postendoscopic sphincterotomy bleeding probabilities. 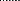, apparent; 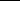, bias‐corrected; 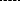, ideal.

**Figure 2:**
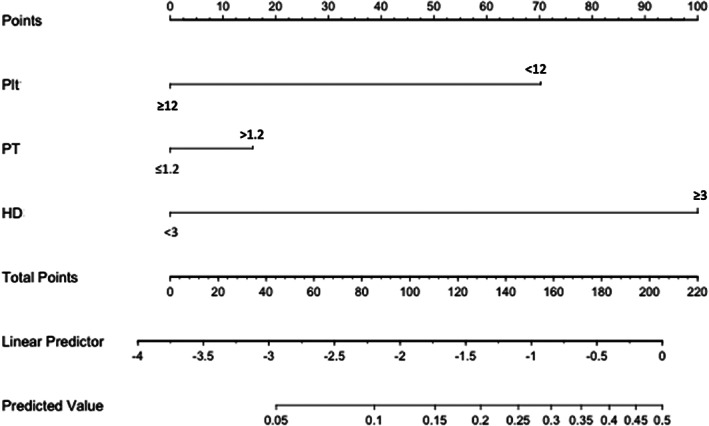
Nomogram depicting the multivariate impact of each variable in our logistic regression model.

The results of our multivariate analysis, including platelet count (<12, ≥12 × 10 000/mL), PT‐INR (≤1.2, >1.2), and HD duration (<3, ≥3 years), are shown in Table 3 (Odds ratio [95%CI]: 0.277 [0.098–0.782], 1.330 [0.381–4.660], and 6.220 [0.779–49.600], respectively). Receiver operating characteristic (ROC) analysis for our logistic regression model found that this model had an area under the curve (AUC) of 0.715 (95% CI, 0.609–0.822). Figure 1 shows the calibration plots for the probabilities of post‐ES bleeding. The 45° line represents the ideal predictions, and this plot shows enough similarity between the actual and the estimated probabilities of post‐ES bleeding rate. Based on these results, we developed a nomogram that visually depicts the multivariate impact of each variable within the logistic regression model (Fig. 2).

### *Secondary outcome measures*

Results for our secondary outcome measures are summarized in Table 4. The mortality rate of HD patients treated with choledocholithiasis was 5.6% (7/123); however, no post‐ES bleeding‐related death occurred. The median interval between ES and post‐ES bleeding was 3.5 days. In addition, delayed bleeding (a delay of 48 h) was observed in 75% (15/20) and rebleeding in 45% (9/20) of all cases.

**Table 4:** Secondary clinical parameters and patient course

Factor	Group	*n* (%)/median [range]			*P*‐value
		Overall	Post‐ES bleeding		
			−	+	
*n*		123	103 (83.7)	20 (16.3)	
Clinical outcome (%)	Death	7 (5.6)	5 (4.8)	2 (10.0)	0.315
	Recovered and discharged	116 (94.4)	98 (95.2)	18 (90.0)	
The cause of deaths (%)	Cholangitis	1 (0.8)			
	Other causes	6 (4.8)			
The duration of the hospitalization period (days)		10 [2–378]	10 [2–378]	19.5 [8–143]	0.001
ERCP related adverse events (%)	Bleeding	20 (16.1)			
	mild	3			
	moderate	5			
	severe	12			
	Pancreatitis	4 (3.2)			
	mild	4			
	Perforation	3 (2.4)			
	Cholecystitis	2 (1.6)			
	Mucosal laceration	1 (0.8)			
The interval between the ES and bleeding (days)				3.5 [1–11]	
	≤2			5 (25.0)	
	3~6			12 (60.0)	
	7≤			3 (15.0)	
The initial hemostasis procedures (%)	Ablation			2 (10.5)	
	Clipping			4 (21.1)	
	EMS			1 (5.3)	
	HSE			6 (30.0)	
	HSE + Ablation			5 (26.3)	
	HSE + Ablation + Clipping			1 (5.3)	
	No intervention			1 (5.3)	
The total number of bleeding				1 [1–8]	
	1			11 (55.0)	
	2~4			5 (25.0)	
	5≤			4 (20.0)	
The total blood transfusion requirement				7 [0–30]	

EMS, expandable metallic stenting; ERCP, endoscopic retrograde cholangiopancreatography; ES, endoscopic sphincterotomy; HSE, hypertonic saline epinephrine solution.

## Discussion

To date, this study is the largest retrospective multicenter study on the rate of post‐ES bleeding in HD patients. Key results were as follows: (i) Post‐ES bleeding occurred in 16.3% (20/123) of HD patients, which is higher than the frequency of post‐ES bleeding in the general population; (ii) cut‐off platelet count, PT‐INR, and HD duration values for increased post‐ES bleeding risk in HD patients were identified; and (iii) a novel predictive model of post‐ES bleeding risk in HD patients was developed.

Regarding risk factors for post‐ES bleeding, a platelet count exceeding 50 000–80 000 and a PT‐INR of <1.2 are generally safe in the general population.^14^ However, in the current study, a platelet count of <120 000 was a strong risk factor for post‐ES bleeding based on univariate and multivariate analyses. This may be attributable to the influence of uremia‐induced platelet dysfunction (due to reductions in the aggregation abilities and adhesiveness of platelets) in HD patients.^11^ On the other hand, there was no significant difference in analysis by PT‐INR binarization. However, the groups showed a trend that presented an increase in the bleeding rate with the increase in PT‐INR; therefore, a small number of the group of PT‐INR prolongation was presumed to cause no significant difference. As there is no direct relationship between HD and PT‐INR prolongation, PT‐INR of <1.2 might be a risk factor in the HD group as in the general population. This was why the cut‐off value of PT‐INR was set to 1.2.

Concerning mean HD duration, there was no significant difference between patients with (7.60 years) and without (6.74 years) post‐ES bleeding. This is in contrast to a previous report where mean HD duration was 19.5 and 6 years in patients with and without post‐ES bleeding, respectively (*P* = 0.029).^9^ This discrepancy could be due to sampling bias; while the previous report included only 21 patients who underwent ES, our study included 123 patients. However, additional analysis showed that post‐ES bleeding incidence was significantly higher in patients with a mean HD duration of ≥3 years than in those with a mean HD duration of <3 years (*P* = 0.025). This suggests that there is a nonlinear correlation between post‐ES bleeding and the duration of HD.

Antithrombotic therapy has also been reported to be an important risk factor for post‐ES bleeding.^15^ However, we found no significant differences in the antithrombotic drugs administered between patients with and without post‐ES bleeding. This might be because all dialysis patients use anticoagulants during dialysis, whether or not they receive oral antithrombotic treatment. However, in this study, the number of patients with inadequate withdrawal period is small and needs further investigation. On the other hand, we did not find evidence for the superiority of NM (a synthetic serine protease inhibitor with a short half‐life) or anticoagulants primarily by inhibiting factor Xa (LMWH) in post‐ES bleeding prevention in HD patients. This is a novel finding that contradicts the current general preference (despite the lack of evidence) for NM or LMWH in HD patients undergoing invasive procedures. These results suggest that the antithrombotic agent administered to patients do not affect their outcome.

The three risk factors above were incorporated into our predictive model, which consisted of six risk groups (i.e. lower‐ and higher‐risk groups for platelet count, PT‐INR, and HD duration). Despite its simplicity, our model has good predictive ability with an AUC of 0.715. Although our model tends to underestimate the risk for low‐risk patients, it is more important to identify high‐risk patients in clinical practice. Hence, it is conceivable that our nomogram is useful in a clinical setting. For example, our nomogram could be used to identify HD patients undergoing treatment for choledocholithiasis who are at an elevated risk of post‐ES bleeding. This would allow the identification of patients who require more careful observation of their clinical course. For such patients, it might be preferable to perform endoscopic papillary balloon dilation (EPBD) instead of ES if the bile duct stone is small (<9 mm).^16^ However, Tsai *et al*. reported that EPBD resulted in fewer post‐ES bleeding events than EST in the non‐HD population (0.75% *vs* 2.26%; *P* = 0.049), but it failed to provide a reduction in the HD population (8.70% *vs* 8.33%; *P* = 0.484).^17^ Therefore, this requires verification by further studies.

In the general population, approximately one‐half of post‐ES bleeding occurs immediately after ES.^18^, ^19^ However, our study revealed that only 25% of post‐ES bleeding occurred within 48 h in HD patients; a delay of 48 h to 11 days was observed in the other patients. Moreover, in cases where post‐ES bleeding had already occurred, the rebleeding rate (45%: 9/20) was higher than that of past reports (5.9%: 8/136 and 27.0% 4/30),^10^, ^20^ and repeat hemostasis is often required, with delayed wound healing and prolonged bleeding likely caused by factors such as malnutrition, peripheral circulatory failure, and immunodeficiency. To prevent additional tissue injury during hemostasis in HD patients who are at greater risk of bleeding, covered self‐expandable metal stent placement might be useful.^21^, ^22^

Despite the insights provided by our study, there are some limitations to consider. First, some candidates were excluded from this study because of deficiencies in their available data. Therefore, this study might have been affected by sampling bias. Second, external validation was not performed for our predictive model because this study was conducted in large hospitals only in Japan. Third, this study was retrospective in nature and had no control group. Nevertheless, it was adequate enough to be able to predict the risk of post‐ES bleeding risk in HD patients.

In conclusion, we found that post‐ES bleeding occurred with higher probability in HD patients than in the general population. We also succeeded in constructing a predictive model of post‐ES bleeding in HD patients, which has the potential to be useful in clinical practice. Possible future studies include external validation of this predictive model and a randomized controlled study to determine whether ES or EPBD is preferable in HD patients.

## Declaration of conflict of interest

The authors declare that there are no conflicts of interest associated with this article.
